# Trauma burden in Tanzania: a one-day survey of all district and regional public hospitals

**DOI:** 10.1186/s12873-017-0141-6

**Published:** 2017-10-13

**Authors:** Hendry R. Sawe, Juma A. Mfinanga, Khalid R. Mbaya, Phillip M. Koka, Said S. Kilindimo, Michael S. Runyon, Victor G. Mwafongo, Lee A. Wallis, Teri A. Reynolds

**Affiliations:** 1 Department of Emergency Medicine, Muhimbili University of Health and Allied Sciences, P.O. Box 65001, Dar es Salaam, Tanzania; 2 Department of Emergency Medicine, Muhimbili National Hospital, Dar es Salaam, Tanzania; 3 Emergency Medicine Department, Al-Zahra Hospital Sharjan, Sharjan, United Arab Emirates; 4 Department of Emergency Medicine, Carolinas Medical Center, Charlotte, NC USA; 5 Division of Emergency Medicine, University of Cape Town, Cape Town, South Africa; 6 Emergency and Trauma Care Program, WHO, Geneva, Switzerland

**Keywords:** Trauma burden, Injured patients, Road traffic crash, Emergency care

## Abstract

**Background:**

Trauma contributes significantly to the burden of disease and mortality throughout the world, but particularly in developing countries. In Tanzania, there is an enormous research gap on trauma; the limited data available reflects realities in cities and areas with moderately- to highly-resourced treatment centers. Our aim was to provide a description of the injury epidemiology across all of Tanzania. Our data will serve as a basis for future larger studies.

**Methods:**

This is a subgroup analysis of a cross-sectional, prospective study of the clinical epidemiology of patients presenting at all public district and regional hospitals in Tanzania. The study was conducted between May 2012 and December 2012. A team of emergency doctors used a purpose-designed data collection sheet to gather the demographic and clinical information of all patients presenting during the day-site visit to each hospital. Descriptive statistics, including means, standard deviations, medians, and ranges are reported.

**Results:**

A total of 5227 patients were seen in 24-h period in 105 (100% response rate) district (or designated district) and regional hospitals in mainland Tanzania. Of these patients, 508 (9.7%) presented with trauma-related complaints. Among patients with trauma-related complaints, 286 (56.3%) were male, and the overall median age of 30 (interquartile range of 22–35) years. Road traffic crash was the most common mechanism of injury, accounting for 227 (44.7%) complaints. Open wounds and bone fractures were the two most frequent diagnoses, with a combined 300 (59%) cases. Most of the patients - 325 (64%) - were discharged, 11 (2.2%) went to operating theatres and 4 (0.8%) of patients died while receiving care at the acute intake areas.

**Conclusions:**

Trauma-related complaints constitute a substantial burden among patients seeking care in acute intake areas of hospitals across Tanzania. There is a need to develop, implement and study systems that can support the improvement of trauma care and optimize outcomes of trauma patients.

## Background

Trauma contributes significantly to the burden of disease and mortality throughout the world, but particularly in developing countries [1–4]. Trauma remains a leading cause of death and disability particularly in paediatric and adolescent populations worldwide. Despite the fact that the quality of the data available from most developing countries is relatively poor, the majority of the past efforts at quantifying the global burden of disease [1, 5, 6] have convincingly established that trauma contributes approximately 10% to global mortality, and 12% to global morbidity. Currently, injuries through Road traffic crash (RTC) are ranked ninth globally among the leading causes of disability adjusted life years lost [7]. It is estimated that 1.2 million people are killed in RTCs each year and as many as 50 million are injured globally [8]. Increasing motorization in many developing countries has seen deaths due to RTCs surge to the degree that such injuries are projected to become the third most important health problem by 2020 [9, 10].

In Africa, injuries rank among the top ten mortality causes, and claim more lives than tuberculosis, malaria and HIV combined [11]. In addition, for every death, there are thousands of non-fatal injuries which, though likely under-reported, result in serious impairment that might be preventable with timely treatment [1, 12]. According to the 2008 WHO injury prevention report, most of the injury burden in the world rests in low-income and middle-income countries, and within these countries, the poor are disproportionately affected [12, 13]. Africa has the highest rate of road traffic injuries resulting in mortality (28.3 per 100,000 population) when corrected for under-reporting, compared with 11.0 per 100,000 in Europe [14].

Tanzania is a low income-country with a population of 55 million people, of whom 80% lives in rural area. The life expectancy is approximately 65 years, and HIV is the leading cause of mortality [15]. There is neither formal pre-hospital system, no trauma care system [16]. Most of the patients receive health care through public system, which is provided through a pyramidal structure from dispensary, health centre, district hospital, regional hospital and consultant hospitals [17]. Tanzania is no exception to that trend, with a high burden of injuries across all age groups, and a notable proportion in children [18]. Being a developing country, Tanzania is also expected to experience the increased trauma, particularly due to the RTC epidemic driven by rapid urbanization, poor infrastructure and the epidemic of motorcycle used as transport. Injuries and death from RTCs are expected to increase if no preventive measures are taken [1]. Many of these injuries are preventable, but targeted prevention efforts are hampered by the limited data [19–22] , which has traditionally focused on the cities and areas with moderately- to highly- resourced centers in the country leaving a gap in understanding the nature of injury and care in the remainder of the country, particularly rural areas. This poses a unique challenge in understanding the real epidemiology of trauma in Tanzania, given majority of Tanzanians live in rural areas [23]. In this study, we sought to provide a pilot description of the injury epidemiology across all of Tanzania by undertaking a 24-h period cross-sectional prospective audit of all public district and regional hospitals in Tanzania. We believe this data will serve as a basis for future larger studies aiming at secondary prevention of trauma-related illness across Tanzania and broader Sub-Saharan Africa.

## Methods

### Study design

This is a subgroup analysis of a larger cross-sectional, prospective study, which described the clinical presentation, investigations, procedures and diagnoses of patients presenting to public district and regional hospitals in Tanzania. The larger cross-sectional, prospective study enrolled a consecutive convenience sample of all patients presenting during the randomly selected daylong clinical shift to each hospital. This study was part of Tanzania Emergency Care Capacity Site Survey (TECCSS) project, which aimed at evaluating three main components of emergency care: the disaster preparedness, equipment availability and disease burden in all district and regional hospitals. The TECCSS project was conducted between May 2012 and December 2012.

### Study setting

All district and regional hospitals in Tanzania mainland were included in this audit. Mainland Tanzania (excluding the island of Zanzibar) at the time of this study had 25 regional and 80 district (or designated district) hospitals. The regional hospitals are expected to receive referral of patients from district hospitals and hence offer an expanded range of care and more specialty services than district facilities.

### Study protocol

The data collection was overseen by one of the five authors (PM, HS, JM, KM and SK), who are all emergency physicians. The researchers were randomly divided into different geographical and political areas of Tanzania based on locations of regional and district hospitals. A purpose-designed data collection sheet was used and the data collectors asked treating providers to complete de-identified data sheets on all patients who presented within that period with the assistance of the researcher. Information gathered included basic demographics, clinical presentations, investigations done (example: imaging and laboratory work), procedures done, clinical management including medications, and disposition. Data not gathered as the patient moved through the facility were collected later the same day from patients’ medical records.

### Data analysis

The study data were transferred from the hand-written data forms into an Excel database (Microsoft corporation, Redmond, WA) and analyzed with SAS (version 9.3, SAS Institute Inc., Cary, NC, USA). Procedure, frequency and univariate functions of SAS were performed to check for any outliers and clean the dataset. Descriptive statistics, including means, standard deviations, medians, and ranges were calculated.

### Ethics approval

The study protocol was reviewed and approved by the Institutional Review Board of the Muhimbili University of Health and Allied Sciences (MUHAS) and Ministry of Health issued a permission to survey all of the hospitals.

## Results

### Hospitals surveyed

We surveyed all 105 district and regional hospitals across mainland Tanzania, and had a 100% response rate. Of these hospitals, 80 (76.2%) were district (or designated district) hospitals, and 25 (23.8%) regional hospitals. The hospitals have been divided in geo-political zones to cover the whole country: There are 14, 10, 10, 19, 10, 6, and 11, district (or designated district) hospitals in the Lake, Eastern, Western, Northern, Southern, Central and Southern Highland zones respectively. Each of 25 political regions had one hospital, and those zones had 3,6, 3, 4, 3, 2, and 4, regional hospitals respectively. Only 11 (10.5%) hospitals had a dedicated 24-h acute intake area with a dedicated doctor on duty to tend to emergencies.

### Patients demographics

A total of 5227 patients were seen in all hospitals. Of these, 508 (9.7%) patients presented with trauma-related complaints. Most 286 (56.3%) of these patients were male, and the overall median age among all patients was 30 years (interquartile range 22 to 35 years). The Eastern zone 151 (29.7%) and southern Western zone 44 (8.7%) had the highest and lowest number of patients per zones respectively (Table 1)**.**

**Table 1:** Patients’ demographics

Demographics	Number (*N* = 508)	Percentage (%)
Sex		
Male	286	56.3
Female	222	43.7
Age Group		
< 5 Years	24	4.7
5–15 years	95	18.7
16–55 years	333	65.6
> 55 years	56	11.0
Patients by Region^a^		
Eastern Zone	151	29.7
Northern Zone	68	13.4
Lake zone	96	18.9
Southern Zone	50	9.8
Central Zone	50	9.8
Southern Highlands zone	49	9.6
Western Zone	44	8.7

^a^The number of patients per 100,000 populations is 2.0,1.1,1.5,1.3,1.5,1.0 and 0.7 for eastern, northern, lake, southern, central, southern highlands and western zone respectively

### Mechanism of injury

RTC 227 (44.7%) represented the most common mechanism of injury, while poisoning and drowning represented the least common mechanisms (Table 2)**.**

**Table 2:** Mechanism of injury

Mechanism	N = 508	% (95 CI)
RTC	227	44.7 (40.4–49.0)
Fall	98	19.3 (15.9–22.7)
Assault	94	18.5 (15.1–21.9)
Animal bite	55	10.8 (8.1–13.5)
Burn	18	3.5 (1.9–5.1)
Others^a^	16	3.1 (1.6–4.6)

^a^Poisoning, drowning, foreign body ingestion

### Distribution of injury mechanism by age group

The overall distribution of injury mechanisms was significantly different across the age groups (*p* < 0.001). Burns and falls were the most common mechanisms among patients under 5 years and 5–15 years of age, respectively, while RTCs were most common among those in the groups of patients 16–55 years and above 55 years of age (Table 3)**.**

**Table 3:** Distribution of injury mechanism by age group*

AGE GROUP: Year	< 5	5–15	16–55	> 55
n	24	95	333	56
RTC (%)	6 (25.0)	22 (23.2)	179 (53.8)	20 (35.7)
Fall (%)	4(16.7)	35 (36.8)	42 (12.6)	17 (30.4)
Assault (%)	1 (4.2)	17 (17.9)	72 (21.6)	4 (7.1)
Burn (%)	8 (33.3)	4 (4.2)	6 (1.8)	0
Animal bite (%)	5 (20.8	13 (13.7)	31 (9.3)	6 (10.7)
Others^a^ (%)	0	4 (4.2)	3 (0.9)	9 (16.1)

*p < 0.001 for the overall distribution of injury mechanisms by age group

^a^Poisoning, drowning, foreign body, other penetrating and other blunt injuries

### Top diagnosis

Open wounds and bone fractures were the top two most frequent diagnoses, accounting for 59% of the top ten diagnoses, while snake-bite 4 (0.8%) was the least frequent diagnosis (Table 4)**.**

**Table 4:** Top diagnosis

DIAGNOSIS	*N* = 508	% (95CI)
Open wounds of head, limbs, neck or trunk	183	36.0 (31.8–40.2)
Bone fracture	117	23.0 (19.3–26.7)
Head injury	49	9.6 (7.0–12.2)
Superficial Contusion	41	8.1 (5.7–10.5)
Dog/cat bite	39	7.7 (5.4–10.0)
Polytrauma	28	5.5 (3.5–7.5)
Other Injuries	23	4.5 (2.7–6.3)
Burn	18	3.5 (1.9–5.1)
Visceral injury	6	1.2 (0.3–2.2)
Snake bite	4	0.8 (0.03–1.6)

### Patient disposition

Most of the patients 325 (64%) were discharged, 11 (2.2%) of patients were channelled straight towards the operating theatres, and 0.8% of patients died while receiving care at the acute intake areas (Table 5)**.**

**Table 5:** Patients’ disposition

DISPOSITION	N = 508	% (95 CI)
Discharged	325	64.0 (59.8–68.2)
Admitted	139	27.4 (23.5–31.3)
Referred	21	4.1 (2.4–5.8)
Theatre	11	2.2 (0.9–3.5)
Unknown	8	1.6 (0.5–2.7)
Died	4	0.8 (0.03–1.6)

## Discussion

Our study represents one of the most comprehensive assessments of one-day trauma burden presenting to acute intake areas in districts and regional public hospitals of Tanzania. Most of the previous studies [19, 20, 24] had focused on the cities and areas with moderately- to highly-resourced hospitals, which left a gap in understanding the nature of injury and care in areas with different socio-economic settings. This study involved those areas but also many places not previously studied providing much-needed foundational knowledge of Tanzania’s trauma burden.

Trauma-related complaints accounted for about 10% of the overall complaints of patients presenting to acute intake areas of the health facilities in Tanzania. Middle aged males were the most affected group in our study population, consistent with previously published literature in Tanzania and Sub-Saharan Africa [22, 25–27]. Open wounds of the head, limbs, neck or trunk and bone fractures accounted for over 50% of the diagnoses recorded, highlighting the challenge of caring for patients especially in hospitals situated in regions and districts further away from the single fully equipped trauma care hospital in the country [28]. While trauma from RTCs was the most common mechanism of injury in all the patients, of interest was the finding that more than 10% of patients presented with injuries from animal bites, in particular dog and cats. This finding highlights an often-unrecognized mechanism that has not been well documented in the past studies, which have primarily focused on RTCs. The significant role of animal bites found in this study is a major issue in Tanzania and most of the developing world due to the fact that few domestic animals have been vaccinated [29]. Animal bites in these areas pose a high risk for transmission of rabies.

The trauma distribution patterns within different age groups differ from those observed in developed countries [30, 31], with most patients below the age of five in our study presenting with injuries related to burns. We believe the same socioeconomic factors observed in previous studies in Tanzania contributed to the observed high proportion of children with burns [20, 32]. The pattern of RTC-related injuries by age was consistent with studies from both high- and low-income countries [33–36]. Overall, 30% of all patients were either admitted, referred to higher levels of care or went to theatre. While this study was not designed to comment on the injury severity of the trauma patients, these results suggest a high level of complexity or severity of injuries as well as a possible lack of resources at these sites. The overall mortality in the acute intake areas was less than one-percent. We believe that our study likely under represents the true mortality from trauma given that we did not follow these patients beyond the acute intake area care.

Beyond the huge and diverse trauma burden, another major finding of our study was that only 10% of hospitals had a dedicated 24-h acute intake area with a dedicated doctor on duty to provide care for patients presenting with emergencies. This is a substantial gap in access to care for patients with trauma-related emergencies that may cause a delay in appropriate treatment. While our study was not designed to identify the reasons for this lack of dedicated 24-h acute intake areas, the observed gap might be a result of combination of resource-related factors and the infancy of emergency medicine as a dedicated discipline in Tanzania [16]. Prior studies in Tanzania have documented global lack of emergency equipment and supplies in most of the acute care intake areas of various levels of hospitals [17].

Over half of the trauma-related complaints were reported in the hospitals of the Eastern and Western zones of Tanzania. These zones have regions with both the highest populations and fastest-growing economies, including rapid urbanization and motorization [23]. While these zones accounted for the highest burden in our studies, the trauma care systems in most of these places are still under developed. There is only one full-capacity trauma care centre in Tanzania [28], that is located in the Eastern zone, which had the highest proportion trauma compared to other zones. All patients needing advanced trauma and neurosurgical interventions are dependent on this fully equipped trauma centre. Most patients in lower levels of care (district and regional hospitals) are dependent on clinical officers and assistant medical officers [17]. Since these personnel are not trained to provide advanced trauma and neurosurgical interventions, most of such patients will require referral to higher levels of care, most often to the only fully equipped trauma centre in Tanzania.

This study provides a description of a one-day trauma burden among patients seeking emergent care in hospitals across Tanzania. As expected, there is a high burden of trauma in both rural and urban areas. While we have provided pilot data to help guide policy and resource allocation, the study has highlighted areas of further research. We propose that the priorities should be given to supporting the implementation of 24-h emergency care units in all districts and regional hospitals so as to support the provision of life saving resuscitation to trauma patients. Furthermore, future research should focus on implementing a standardized trauma documentation system across the country, to further characterize the risk factors, clinical care, referral patterns and outcomes in order to identify areas of improvement and optimize outcomes.

### Limitations

This was a one-day study that was collected on different days and by different health care professionals. In addition, our results reflect reported and recorded patient characteristics, which may be incomplete. We did, however, include all district and regional hospitals, and gather data in real time during on site visits to each facility, which we believe increases the accuracy of this cross-sectional characterization.

## Conclusion

Trauma-related complaints constitute a substantial burden among patients seeking care in acute intake areas of hospitals across Tanzanian. There is a need to develop, implement and study systems that can support the improvement of trauma care and optimize outcomes of such patients.
